# Complications and Clinical Outcomes Following Modified Endoscopic Denker's With Pyriform Aperture Preservation: A Prospective Analysis

**DOI:** 10.1002/alr.70200

**Published:** 2026-06-12

**Authors:** Yasser Almansour, Lane Donaldson, Khaled Kashlan, Swapna Vemuri, Jun Jin, John R. Craig

**Affiliations:** ^1^ Department of Otolaryngology‐Head and Neck Surgery Michigan State University College of Human Medicine East Lansing Michigan USA; ^2^ Department of Otolaryngology‐Head and Neck Surgery Henry Ford Health Detroit Michigan USA; ^3^ Department of Ophtalmology Henry Ford Health Detroit Michigan USA; ^4^ Department of Public Health Sciences Henry Ford Health System Detroit Michigan USA; ^5^ Department of Epidemiology and Biostatistics Michigan State University East Lansing Michigan USA

**Keywords:** endoscopic Denker's, endoscopic medial maxillectomy, endoscopic sinus surgery, epiphora, hypesthesia, inverted papilloma

## Abstract

**Background:**

The modified endoscopic Denker's (MED) approach provides access to all maxillary sinus (MS) walls for resection of various MS and retromaxillary pathologies, but the anteromedial maxilla has historically been resected. This study determined complication rates and outcomes following MED with pyriform aperture (PA) preservation (MEDPAP).

**Methods:**

A prospective study was conducted on all adults who underwent MEDPAP for different MS or retromaxillary pathologies over 7.5 years. The following complications were collected at all postoperative visits: epiphora, hypesthesia or complete numbness (teeth, upper lip, lateral nasal sidewall, and cheek), crusting, nasal valve collapse (NVC), and empty nose syndrome 6‐item questionnaire (ENS6Q). Complications and disease recurrence rates were determined at a minimum 1 year for most complications and outcomes and 2 years for hypesthesia if hypesthesia had not resolved.

**Results:**

Of 52 MEDPAP patients, median age was 58.5 years; 55.8% were male. Of 34.6% who experienced epiphora postoperatively, 7.7% persisted. When epiphora resolved, it did so by median 63.0 days. Sites of initial postoperative hypesthesia or numbness included teeth (23%–50%), upper lip (42%), nasal (22%), and cheek (30%). Hypesthesia resolved in 88%–100% of subsites by median 89.5–167.0 days. Hypesthesia persisted in 12% of patients (mainly anterior teeth). Crusting resolved in 97.8% by median 91.0 days. No patients developed NVC, and median final ENS6Q = 0.5 (range = 0–8). Non‐tumor disease and tumor recurrence rates were 0%.

**Conclusions:**

MEDPAP was 100% successful at managing benign and select malignant MS pathologies during the study period. MEDPAP patients frequently experienced temporary epiphora and dentofacial hypesthesia or numbness that resolved by 2–6 months postoperatively. Epiphora and hypesthesia or numbness persisted in 7.7% and 12% of patients, respectively. No patients developed NVC or ENS.

## 1 Introduction

The modified endoscopic Denker's (MED) approach provides access to all maxillary sinus (MS) walls, and has been highly effective for managing various benign and some malignant pathologies. MED is a type of medial maxillectomy that involves resecting the entire medial MS wall, including complete inferior turbinate resection and nasolacrimal duct (NLD) transection. Anteriorly, the MED has classically involved removing varying extents of the anteromedial maxilla, including the bone of the pyriform aperture (PA) [1]. While the MED yields high treatment success rates, especially for MS inverted papilloma (IP), postoperative complications like epiphora, dentofacial hypesthesia, and nasal valve collapse (NVC) can occur.

Largest MED studies to date have reported persistent epiphora in 7%–12% of patients [2, 3, 4]. Rates of long‐term dentofacial hypesthesia, most commonly from anterior superior alveolar nerve (ASAN) injury, have been reported in up to 30% of patients [2, 4], but other branches from the infraorbital nerve (ION) can also be damaged [2, 3]. These studies have not reported exact subsites affected by hypesthesia, nor the times to resolution of epiphora and hypesthesia in those whose symptoms resolve. While uncommon, NVC can also occur after MED due to disruption of ligamentous support of the lower lateral cartilages. Lastly, empty nose syndrome (ENS) could theoretically develop after MED, though this has not been reported. Notably, the previously mentioned series performed MEDs with removal of the anteromedial maxilla and PA [2, 3]. The purpose of this prospective study was to determine the frequencies of long‐term complications and disease recurrence following MED with PA preservation (MEDPAP).

## 2 Methods

A prospective observational study was conducted on consecutive adult patients who underwent a unilateral MEDPAP for a variety of MS or retromaxillary pathologies by the senior author (JRC) from October 2016 through March 2024. Approval was obtained by the Henry Ford Health Institutional Review Board.

All patients underwent a unilateral MEDPAP, also described as a Type C endoscopic medial maxillectomy (EMM) by Schreiber et al. [1], or Type 3A EMM by Turri‐Zanoni et al. [4]. Each MED began with complete inferior turbinectomy followed by removal of the entire medial MS wall from the palatine bone posteriorly to the PA anteriorly (i.e., PA preservation) and from the MS roof superiorly to the nasal floor inferiorly. A 30,000 RPM, 70 tapered diamond bur was used for medial MS wall drilling (Medtronic, Minneapolis, MN). MS mucosa was removed in accordance with pathologies. For retromaxillary pathologies, posterior MS wall mucosa was only removed over the bone being removed for access to the pterygopalatine and infratemporal fossae (PPF, ITF). For invasive fungal sinusitis, all MS mucosa was diseased and removed. For benign tumors, the mucosa at or around their origins was removed, and when hyperostotic foci were present at tumor attachment sites, those were drilled down flush to the MS walls [5]. When tumor originated from bone along the ION, bone was carefully drilled down until thhe bone appeared smooth, with care to preserve at least some bone over the ION. When it was difficult to discern normal from neoplastic mucosa, mucosa would be removed until confident of clear margins. At case conclusion, the NLD was transected obliquely just inferior to its exit from the lacrimal sac, and no lacrimal stenting was performed. All cases were performed with a combination of 0°, 30°, and 70° nasal endoscopes. Figure 1 shows radiographically and endoscopically how each MEDPAP was performed.

**FIGURE 1:**
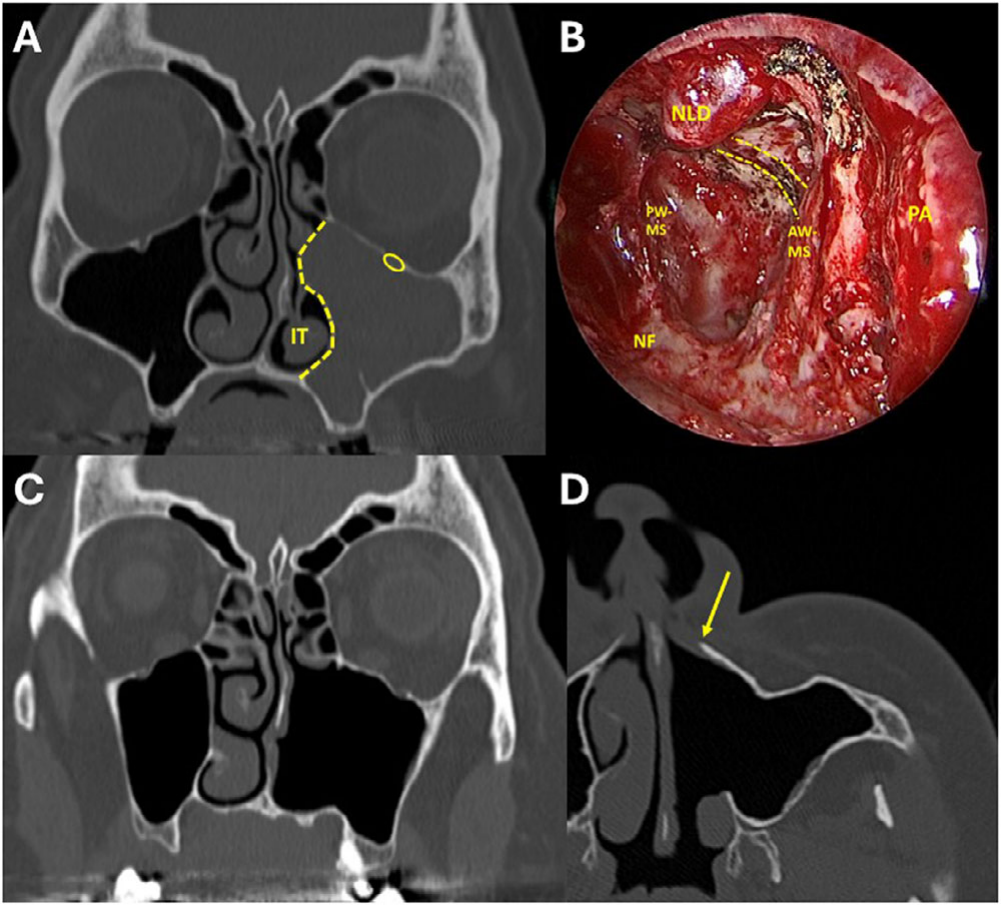
Example of the modified endoscopic Denker's with pyriform aperture preservation (MEDPAP) for a left maxillary sinus inverted papilloma. (A) Coronal bone‐window CT showing left maxillary sinus (MS) complete opacification. There was no focal hyperostosis, and the inferior bony septation was not the origin. The tumor was attached along nearly the entire superior aspect of the anterior wall of the MS (AW‐MS), including the bone on and around the infraorbital nerve (ION, yellow circle). The MED involves removing the inferior turbinate (IT) and entire medial MS wall (yellow dotted line), and in this study, the opening was only carried anteriorly to the PA. (B) 70° nasal endoscopic view of the left MS after MEDPAP, with an opening inferiorly to the nasal floor (NF) and anteriorly to the PA. Cautery is seen along the tumor's origin along the entire superior aspect of the AW‐MS, including around the ION (outlined along the MS roof with yellow dotted lines). Note the obliquely transected nasolacrimal duct (NLD). Normal mucosa was preserved along the posterior MS wall (PW‐MS). (C–D) Coronal and axial bone‐window CTs showing a widely patent MED cavity with no tumor recurrence 5 years postoperatively (scan obtained for unrelated reasons), with the bone of the PA being intact (yellow arrow).

Clinical data were prospectively collected at preoperative and all postoperative appointments. Demographics, pathologies, primary versus revision surgery, 22‐item Sino‐Nasal Outcome Test (SNOT‐22), and presence of hypesthesia or epiphora were recorded preoperatively. For tumors, tumor origin was noted based on review of the intraoperative recordings by the senior author. Additionally for IP, all computed tomography scans were reviewed by the senior author to record presence of hyperostotic foci. Figure 2 highlights how tumor origin/attachments and focal hyperostoses were coded, based on the MS wall involved, and relationships to the ION and NLD. The following complications were analyzed at all postoperative visits: epiphora, dentofacial hypesthesia, internal and external static and dynamic NVC, ENS 6‐item questionnaire (ENS6Q), and crusting.

**FIGURE 2:**
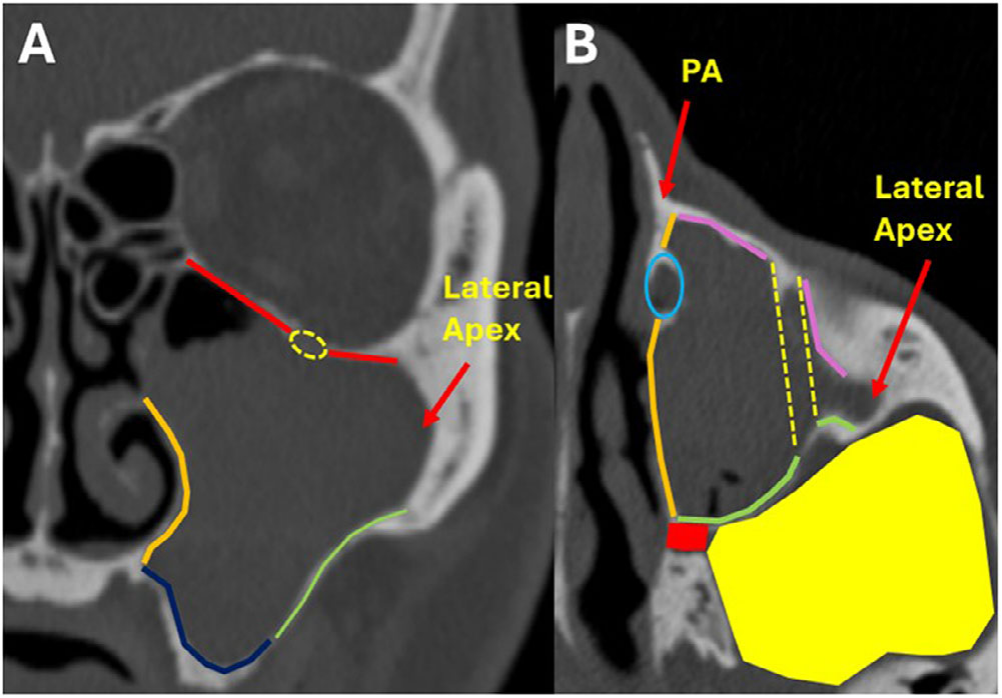
CT‐based coding of maxillary sinus (MS) and retromaxillary tumor origins. (A) Coronal bone‐window CT of left MS, showing the MS roof origins as being medial or lateral to or along the infraorbital nerve (ION, yellow dotted circle), medial MS wall (orange line), inferior MS wall (dark blue line), and posterior MS wall lateral to ION (green line). The lateral apex is highlighted too. (B) Axial CT showing the infratemporal fossa (yellow space), pterygopalatine fossa (red space), medial MS wall (orange lines) anterior or posterior to or at the nasolacrimal duct (NLD, light blue circle), anterior MS wall (purple lines) medial or lateral to or at the ION (yellow dotted lines), posterior MS wall (green lines) medial or lateral to or at the ION. The lateral apex and pyriform aperture (PA) are also highlighted.

Epiphora was assessed by patient report, and if still present at second postoperative appointments around 2–3 months after surgery, patients were referred to an oculoplastic surgeon for evaluation via lacrimal saline irrigation. Dentofacial sensation was evaluated with light fingertip palpation of the cheek, lateral nasal sidewall, upper lip, and individual maxillary teeth ipsilateral to the MEDPAP, and these were compared to the contralateral dentofacial regions. Any patient‐reported reduction in sensation was considered hypesthesia, and complete lack of sensation was considered numbness. Internal and external nasal valves were assessed for static narrowing at rest and dynamic NVC during normal inspiration without nostril occlusion. Endoscopic evidence of MS crusting that was at least partially obstructive to MS outflow, or associated with mucus or purulence, was noted at each visit. For hypesthesia or numbness, patients were followed for minimum 2 years, or until hypesthesia had completely resolved. If persistent, patients were asked if their hypesthesia or numbness was bothersome. Tumor patients were followed for minimum 1 year, though they were encouraged to follow up for at least 5 years [6].

Regarding clinical outcomes, SNOT‐22 change was calculated in patients from preoperative to final postoperative visits. Patency of the MS surgical openings and non‐tumor disease and tumor recurrences were reported at final postoperative visits as well.

Statistical analysis was performed using R version 4.2.2. Descriptive statistics were described with median, interquartile ranges, and ranges for continuous variables, while categorical variables were reported as percentages. Median times to resolution of epiphora, hypesthesia, and crusting were calculated in patients who experienced resolution. Rates of hypesthesia versus complete numbness at different dentofacial subsites were also compared at first and final follow‐up visits using a Fisher's exact test. Note that patients with PPF and ITF tumors were excluded from analyses on time to resolution of both hypesthesia and crusting. Their hypesthesia rates were likely to confound analyses due to direct trigeminal nerve involvement by lesions, and their crusting would likely be less since they had undiseased maxillary sinuses and required substantially less MS mucosal removal. Changes in SNOT‐22 were analyzed with the Wilcoxon rank‐sum test, and a subgroup analysis was performed for IP patients. A temporal analysis was then performed to assess whether MEDPAP‐related complications or clinical outcomes differed significantly between early and late patient cohorts using a Fisher's exact test for categorical variables and Wilcoxon rank sum test for continuous variables. Early and late cohorts were defined as the first half and last half of patients, respectively, during the study period.

## 3 Results

Of 52 MEDPAP patients, median age was 58.5 years, and 55.8% were male. Table 1 shows preoperative demographic and clinical variables. MEDPAPs were performed following prior surgical attempts at resecting the disease in 15/52 patients (28.2%). Of the 15 patients who had prior MS surgery, 46.7% had maxillary antrostomies, 40.0% had modified endoscopic medial maxillectomies (removal of medial MS wall and middle portion of IT, while preserving the NLD), and 13.3% had Caldwell‐Luc antrostomies. Different tumors made up 48/52 pathologies (92.3%), with IP being the most common condition overall (78.9%). Two patients had invasive fungal sinusitis, and two had odontogenic cysts. No patients had hypesthesia preoperatively. One IP patient had epiphora preoperatively that resolved postoperatively after primary MEDPAP.

**TABLE 1:** Preoperative demographic and clinical features in patients who underwent unilateral modified endoscopic Denker's approaches with pyriform aperture preservation. IQR, interquartile range (Q1, Q3).

Variables (*N* = 52)	*N* (%)
Age (median [IQR])	58.5 (49.8, 70.5)
Male sex	29 (55.8%)
Right side	30 (57.7)
Primary surgery	37 (71.2)
Preoperative epiphora	1 (1.9)
Preoperative hypesthesia or numbness	0 (0.0)
Pathologies	
Inverted papilloma	41 (78.9)
Invasive fungal sinusitis	2 (3.8)
Schwannoma	2 (3.8)
Odontogenic keratocyst	2 (3.8)
Odontogenic (not keratocyst)	1 (1.9)
Lobular capillary hemangioma	1 (1.9)
Squamous cell carcinoma in‐situ	1 (1.9)
Epithelial‐myoepithelial carcinoma	1 (1.9)
Leiomyosarcoma	1 (1.9)

Table 2 shows frequencies of tumor origins and locations of hyperostotic foci in IP patients. Origins along the anterior wall were most common, and multifocal origins were noted in 37.5% of tumor patients (18/48). Focal hyperostosis was noted in 26/41 IP patients (63.4%).

**TABLE 2:** Frequencies of different tumor origins along different maxillary sinus (MS) walls in those with tumors seen endoscopically, and frequencies of focal hyperostosis seen on CT in inverted papilloma (IP) patients.

Tumor origin (*N* = 48)	Frequency (*n*/48 [%])	Focal hyperostosis in IPs (*N* = 41)	Frequency (*n*/41 [%])
Anterior at/along ION	11 (22.9)	Anterior at/along ION	8 (19.50%)
Anterior medial (medial to ION)	10 (20.8)	Anterior medial (medial to ION)	6 (14.60%)
Anterior lateral (lateral to ION)	13 (27.1)	Anterior lateral (lateral to ION)	10 (24.40%)
Posterior at/along ION	2 (4.2)	Posterior at/along ION	2 (4.90%)
Posterior medial (medial to ION)	5 (10.4)	Posterior medial (medial to ION)	2 (4.90%)
Posterior lateral (lateral to ION)	5 (10.4)	Posterior lateral (lateral to ION)	4 (9.80%)
Inferior, anterior (anterior to the NLD)	4 (8.3)	Inferior, anterior (anterior to the NLD)	1 (2.40%)
Inferior, posterior (posterior to the NLD)	3 (6.2)	Inferior, posterior (posterior to the NLD)	2 (4.90%)
Superior at/along ION (mid MS roof)	1 (2.1)	Superior at/along ION (mid MS roof)	0 (0.00%)
Superior medial (medial to ION)	5 (10.4)	Superior medial (medial to ION)	0 (0.00%)
Superior lateral (lateral to ION)	0 (0.00%)	Superior lateral (lateral to ION)	0 (0.00%)
Medial at/along the NLD	7 (14.6)	Medial at/along the NLD	1 (2.40%)
Medial anterior (anterior to the NLD)	5 (10.4)	Medial anterior (anterior to the NLD)	
Medial posterior (posterior to the NLD)	4 (8.3)	Medial posterior (posterior to the NLD)	1 (2.40%)
Lateral apex	4 (8.3)	Lateral apex	3 (7.30%)
Diffuse, all walls	7 (14.6)	Diffuse, all walls	2 (4.90%)
PPF	1 (2.1)		
PPF and ITF	1 (2.1)		

Abbreviations: ION, infraorbital nerve; LND, nasolacrimal duct; PPF, pterygopalatine fossa; ITF, infratemporal fossa.

Table 3 shows rates of no hypesthesia, hypesthesia, and complete numbness at first and final follow‐ups postoperatively. Figure 3 shows graphically the median times to resolution of these complications, and Table S1 shows the sample sizes and median times to resolution of each complication. Epiphora was present in 18/52 patients initially (34.6%) and became permanent in 4/52 (7.7%). For those whose epiphora resolved, median time to resolution was 63.0 days. Of the four patients with persistent epiphora, two underwent endoscopic dacryocystorhinostomy (DCR), and the other two deferred.

**TABLE 3:** Frequencies of no hypesthesia, hypesthesia, and complete numbness in patients who did not have retromaxillary schwannomas (*n* = 50), at both first and final follow‐ups. *p*‐values were generated from a Fisher's exact test.

	No hypesthesia	Hypesthesia	Complete numbness	*p*‐value (hypesthesia vs. complete numbness)
**Subsites (first follow‐up; *N* = 50)**				
Cheek	35 (70%)	7 (14%)	8 (16%)	1.000
Lateral nasal sidewall	39 (78%)	3 (6%)	8 (16%)	0.227
Upper lip	29 (58%)	8 (16%)	13 (26%)	0.383
Central incisor	27 (54%)	9 (18%)	14 (28%)	0.405
Lateral incisor	25 (50%)	10 (20%)	15 (30%)	0.424
Canine	28 (56%)	8 (16%)	14 (28%)	0.286
First premolar	32 (64%)	6 (12%)	12 (24%)	0.238
Second premolar	36 (72%)	5 (10%)	9 (18%)	0.424
First molar	37 (74%)	5 (10%)	8 (16%)	0.581
Second molar	38 (76%)	5 (10%)	7 (14%)	0.774
**Subsites (final follow‐up; *N* = 50)**				
Cheek	50 (100%)	0 (0%)	0 (0%)	N/A
Lateral nasal sidewall	50 (100%)	0 (0%)	0 (0%)	N/A
Upper lip	49 (98%)	1 (2%)	0 (0%)	
Central incisor	46 (92%)	3 (6%)	1 (2%)	N/A
Lateral incisor	44 (88%)	5 (10%)	1 (2%)	N/A
Canine	45 (90%)	4 (8%)	1 (2%)	N/A
First premolar	48 (96%)	2 (4%)	0 (0%)	N/A
Second premolar	50 (100%)	0 (0%)	0 (0%)	N/A
First molar	50 (100%)	0 (0%)	0 (0%)	N/A
Second molar	50 (100%)	0 (0%)	0 (0%)	N/A

**FIGURE 3:**
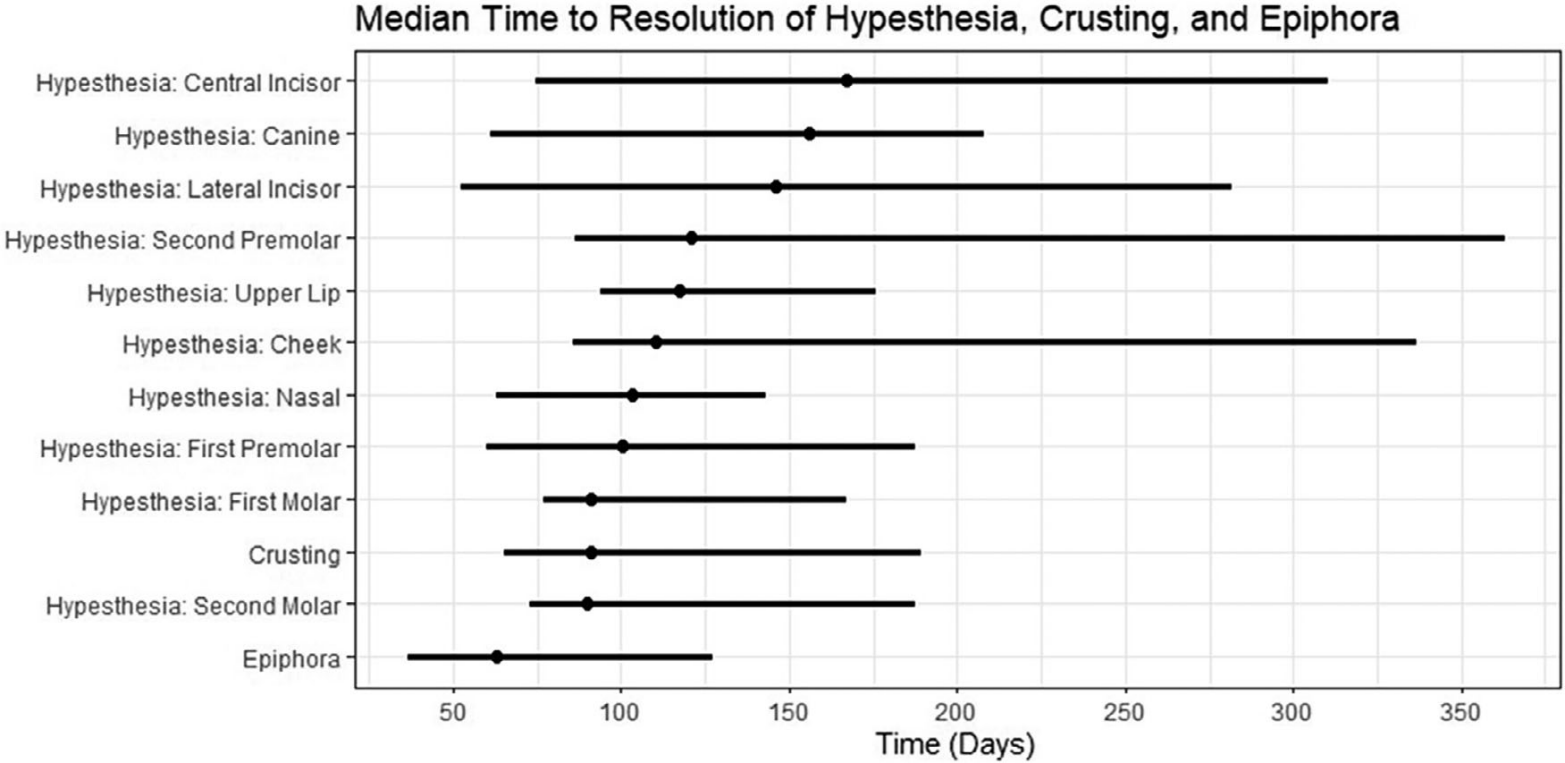
Graphical representation of the median times to resolution of dentofacial hypesthesia, sinonasal crusting, and epiphora when these issues resolved. Error bars represent interquartile ranges (Q1–Q3).

Hypesthesia, or numbness, was initially present in 32/50 patients (64%), with 19/50 (38%) having complete numbness at different subsites, with no significant differences between hypesthesia and numbness at different subsites. Sites of initial hypesthesia or numbness included teeth (23–50%), upper lip (42%), lateral nasal sidewall (22%), and cheek (30%). Hypesthesia of different sites resolved in 88%–100% of patients by a median 89.5–167.0 days. Ultimately at final follow‐ups, 6/50 (12%) had persistent hypesthesia, and 1/50 (2%) had complete numbness of the ipsilateral central and lateral incisors and a canine. Amongst sites of persistent hypesthesia, most common were incisors, canines, and first premolars in 4%–12% of patients at 3.6–5.5 years postoperatively. One patient's upper lip hypesthesia was still present at 2.2 years after surgery. None of these patients reported being bothered by the hypesthesia or numbness at final follow‐ups. Figures 4 and 5 show examples of patients with MS IP with origins on and around the ION, one who experienced no hypesthesia (Figure 4). The other initially had complete numbness of cheek, upper lip, nasal sidewall, and all teeth, but gradually most subsites returned to normal by 8 months postoperatively, except for complete numbness of the central and lateral incisors and canine (Figure 5).

**FIGURE 4:**
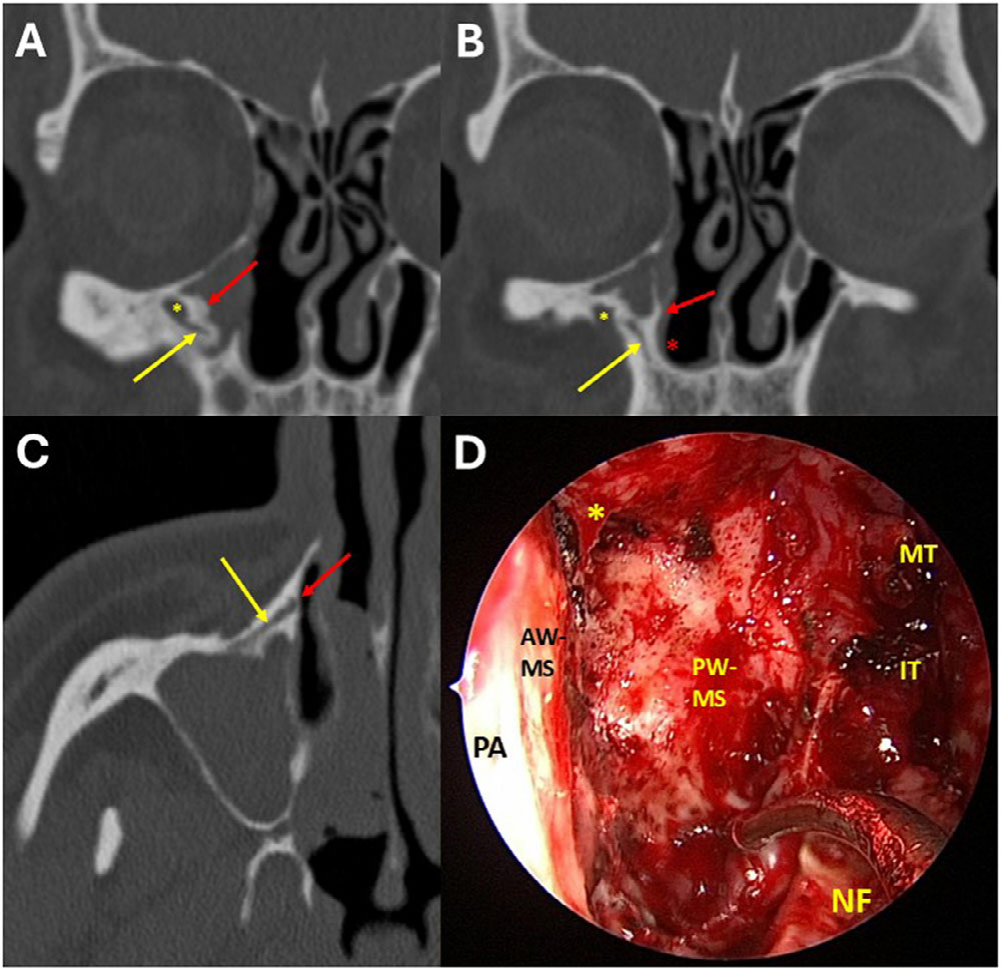
Representative patient who had a right‐sided maxillary sinus (MS) inverted papilloma resected via a modified endoscopic Denker's with pyriform aperture (PA) preservation. (A) Coronal bone‐window CT showing complete opacification of the right MS, highlighting focal hyperostosis (red arrow) over the infraorbital nerve (ION; yellow asterisk), and the horizontal portion of the anterior superior alveolar nerve coursing medially (ASAN; yellow arrow). (B) Another coronal CT showing the vertical portion of the ASAN (yellow arrow) coursing inferolateral to the inferior turbinate insertion (red arrow) and within the bone lateral to the nasal cavity (red asterisk). ION highlighted too (yellow asterisk). (C) Axial CT showing the ASAN (yellow arrow) coursing medially within the anteromedial wall of the MS to the bony edge of the PA (red arrow). (D) Note that 70° nasal endoscopic view of the right MS after MED and complete resection of the tumor, while preserving the PA. Note the hyperostotic tumor origin over the ION (yellow asterisk) had been drilled down carefully, and some cautery had been applied around this area. This patient experienced no hypesthesia anywhere after surgery. AW‐MS, anterior wall of MS; IT, inferior turbinate; MT, middle turbinate; NF, nasal floor; PW‐MS, posterior wall of MS.

**FIGURE 5:**
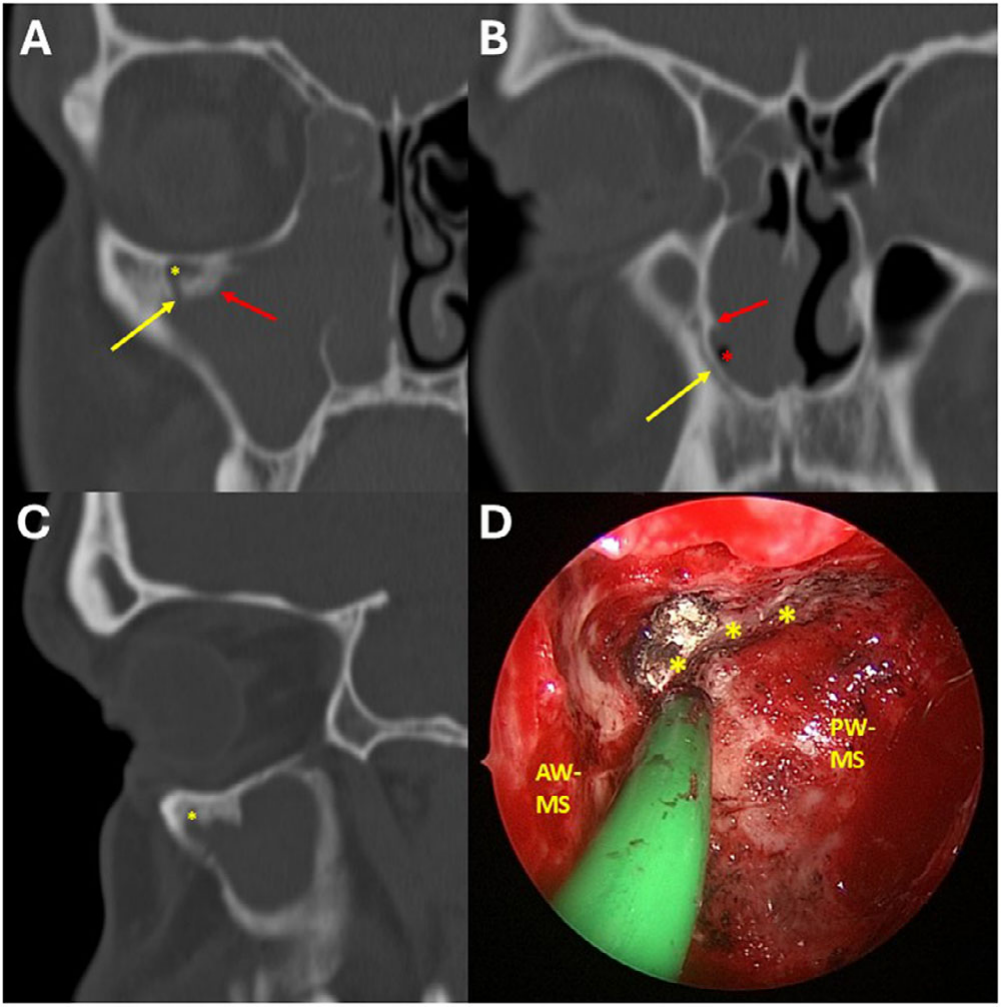
Another representative patient who had a right‐sided maxillary sinus (MS) inverted papilloma resected via a modified endoscopic Denker's with pyriform aperture (PA) preservation. (A) Similar to patient in Figure 4, the coronal bone‐window CT showed complete opacification of the right MS, with focal hyperostosis (red arrow) over the infraorbital nerve (ION; yellow asterisk) and the anterior superior alveolar nerve (ASAN; yellow arrow) coursing inferiorly from the ION. (B) Coronal CT showing the vertical portion of the ASAN (yellow arrow) coursing inferolateral to the inferior turbinate (IT) insertion (red arrow) and nasal cavity (red asterisk). Note that the ASAN coursed very close to the IT insertion and lateral wall of the nasal cavity, placing it at increased risk for direct injury when drilling during the MED. (C) Sagittal CT showing the ION (yellow asterisk) surrounded by hyperostotic tumor origin. (D) Note that 70° nasal endoscopic view of the right MS after complete tumor resection. Note the hyperostotic tumor origin over the ION course (three yellow asterisks) had been drilled down carefully, with cautery applied broadly to this area due to excessive bleeding from the tumor origin. This patient experienced complete right‐sided dentofacial numbness, but all areas resolved completely except for the anterior teeth, presumably due to ASAN transection during drilling near the PA. AW‐MS, anterior wall of MS; PW‐MS, posterior wall of MS.

For crusting, one patient had persistent nasal crusting without purulence at final follow‐up 5.5 years postoperatively following leiomyosarcoma resection without radiation therapy (1.9%). For those whose crusting resolved, median time to resolution was 91 days. No patients developed internal or external nasal valve static or dynamic NVC. Median initial postoperative ENS6Q score was 2.5 (IQR = 1.0, 6.75; range = 0–21), and median final postoperative ENS6Q score was 0.5 (IQR = 0, 1.75; range = 0–8), and the difference was statistically significant (*p* < 0.001; Figure S1).

Regarding other clinical outcomes, in non‐tumor patients (*n* = 4), there were no disease recurrences at final follow‐ups at median 2.8 years (IQR = 1.5, 4.2; range = 1.4–4.6). For tumors (*n* = 48), there was a 0% recurrence at a median 3.6 years (IQR = 1.3, 5.5; range = 1.0–8.3). Also of note for IP patient follow‐up durations, 68.3% had ≥2 years, 56.1% had ≥3 years, and 26.8% had ≥5 years.

All MS surgical openings remained patent at final follow‐ups, and overall median SNOT‐22 reduction was 12.0 (IQR = 4, 40), from 27.0 (IQR = 14, 51) preoperatively to 11.5 (IQR = 1.5, 20.2) postoperatively (*p* < 0.001). Among IP patients, median SNOT‐22 reduction was also 12.0 (IQR = 4.0, 38.5), from 26.0 (IQR = 13.5, 48.5) preoperatively to 6.0 (IQR = 0.0, 18.5) postoperatively (*p* < 0.001).

Tables S2 and S3 show that there were nearly no significant differences between 26 early and 26 late patient cohorts when comparing complication rates, SNOT‐22 changes, and ENS6Q scores at first and final follow‐ups. ENS6Q showed a significant difference between early and late cohorts at first follow‐ups, but this became non‐significant at final follow‐ups. Disease recurrence and NVC rates were too infrequent to compare between cohorts.

## 4 Discussion

MED and MEDPAP provide excellent access for resecting benign MS, PPF, and ITF pathologies, as well as some select malignancies. Prior series on MEDs and MEDPAPs involving MS IP patients have demonstrated 2%–5% tumor recurrence rates [2, 3, 4, 7], substantially lower than prior IP‐related series that have reported 17%–25% MS IP recurrence rates [8, 9]. The current study showed that MEDPAP was at least as effective as MED at 1 year postoperatively, demonstrating a 0% tumor recurrence in this cohort of predominantly IPs, with 70% of tumor patients being followed for at least 2 years postoperatively. Considering the current study's minimum follow‐up duration was similar to prior MED‐related studies with tumor resections [2, 4, 10], tumor resection outcomes with MEDPAP were comparable to MED. However, it is also important to note that only 30% of patients were followed for over 5 years, so delayed recurrences could still occur [6]. Also important, long‐term complication rates were low: 7.7% epiphora, 12% non‐bothersome hypesthesia (mainly anterior teeth), 2% crusting, andno patients developed NVC or ENS.

Prior MED series have demonstrated low rates of epiphora, hypesthesia, and NVC after classic MED with PA removal. Preserving the PA during MED could theoretically lower the rates of both dentofacial hypesthesia and NVC, though the epiphora rate should be equivalent. Schreiber et al. first described in 2016 the MEDPAP‐type approach based on 6 cadaveric dissections but termed it a Type C EMM [1]. Since then, only one study by Turri‐Zanoni et al. has reported clinical outcomes and complications following this extent of EMM, though they termed it a Type 3A EMM [4].

Regarding epiphora, based on series with over 50 patients who have undergone MED or MEDPAP and no lacrimal stenting, about 7%–12% of patients develop epiphora [2, 4, 10]. The current study reinforced these prior studies by demonstrating a 7.7% permanent epiphora rate. Surgeons should be aware that when transecting the NLD, patients are at risk for epiphora, so this approach should only be performed when the MS disease cannot be removed without NLD transection. While prior smaller MED series have suggested lacrimal stenting is unnecessary based on 0% epiphora rates [11, 12], a powered comparative study would be beneficial in the future to determine if lacrimal stenting decreases NLD stenosis rates. Other surgeons have advocated for upfront DCR at the time of NLD transection [13]. While concurrent DCR could be considered, one should also consider the 5%–10% failure rate of primary DCRs [14, 15, 16]. Therefore, due to the low risk of epiphora following NLD transection with MEDPAP (roughly 10% across studies), performing a concurrent DCR could place patients at unnecessary risk since epiphora is unlikely to occur. DCR should be reserved for patients who develop permanent epiphora following NLD transection.

Hypesthesia during MED occurs most commonly from ASAN damage, which supplies at least the anterior maxillary dentition. Turri‐Zanoni et al. performed 249 MEDs (230 PA removal and 19 anterior maxilla removals) and followed patients for 12‐180 months (median 51 months). They reported 10%–30% hypesthesia rates at 6 months but only reported them as either nasal ala or dentition without specifying sites. They also performed 72 MEDPAPs (termed 3A EMM) but did not report hypesthesia rates [4]. Bertazzoni et al. performed 59 MEDs and followed patients for 10–190 months (median 53 months). They reported a 29% rate of hypesthesia of the anterior maxillary dentition but did not report exact teeth. They reported an 8% cheek hypesthesia rate too, but they did not assess the upper lip and lateral nasal sidewall [2]. McCormick et al. performed 58 MEDs with bony PA removal and followed patients for 1–127 months (mean 30 months). Conversely, they reported only a 5% rate of long‐term hypesthesia, with no dental hypesthesia (one upper lip, one palate, one face) [3].

The current study showed that, similar to Bertazzoni et al., nearly all patients with permanent hypesthesia or numbness experienced this in the incisors and canines. Notably, while the majority of patients initially developed hypesthesia or numbness (40% complete numbness) at different dentofacial subsites, the overwhelming majority of these sensory disturbances improved or resolved over time, implying either nerve regeneration or collateral branching from nearby trigeminal branches. Also interesting, about one‐third of patients experienced no postoperative hypesthesia (18/50). This could be due to variable ASAN courses or possibly variable amounts of drilling performed at the most anterior and inferior aspects of the PA. Machado et al. studied relationship of the descending portion of the ASAN to the inferior turbinate's anterior insertion in 50 patients. They found that the ASAN always coursed inferolaterally to the IT insertion, but the distance between the two structures varied [17]. It is possible that assessing preoperative CTs for this relationship could help with ASAN preservation, and future studies could explore this. As an example, when comparing ASAN courses in Figures 4b and 5b, one can see in Figure 5b the ASAN coursing closer to the IT insertion and PA, which could have put it at higher risk of injury during drilling (this patient initially had complete numbness from ASAN injury).

Even with PA preservation in this study, there were still some injuries to the ION or other branches proximal to the ASAN during removal of the MS pathologies. These were more likely neuropraxic thermal injuries from drilling and monopolar cautery used on bone overlying these nerve branches, as evidenced by temporary hypesthesia or numbness in the cheek, upper lip, and lateral nasal sidewall. Reinforcing that most nerve injuries following surgeries of the lateral nasal wall are neuropraxic, Machado et al. recently reported sensory outcomes prospectively following different extents of lateral nasal wall surgery and showed that most injuries are temporary, with most patients’ dentofacial hypesthesias resolving or improving substantially by 3 months postoperatively [18]. Only one patient in the current study experienced long‐term but non‐bothersome upper lip hypesthesia, but this patient had a small ION branch avulsed during tumor removal, which likely caused the permanent deficit. Notably, even that patient regained some function over time, implying some collateral re‐innervation.

Another important point for patients with tumors originating on or around the ION was that a 0% tumor recurrence rate was achieved, and patients had no cheek, nasal, or upper lip hypesthesias as long as nerve branches were not avulsed. Therefore, surgeons should feel comfortable drilling down and cauterizing bone judiciously at tumor origins near or on the ION and its branches. Note in this study that the bone was polished down just enough to be confident that tumor rests within bone had been eliminated. Surgeons do not need to remove all the bone at tumor origins down to nerve sheaths, as this could devascularize or directly avulse the nerve and place it at further injury risk should cautery be needed.

This study built upon prior MED‐related studies to provide important prognostic information for patients with epiphora and hypesthesia. Based on this study, patients can be reassured that roughly 80% of initial epiphora will resolve by 2 months postoperatively (14/18) and that about 80% of hypesthesia will resolve by 3–6 months postoperatively (25/32), with the anterior teeth being the latest to recover. Importantly, patients should know that hypesthesia can take many months to recover, and while the overwhelming majority of patients will recover by 1 year following surgery, some patients can regain sensation even after 1 year postoperatively.

Another reported complication with MED approaches is NVC. With MEDs that include resection of the PA and anteromedial maxilla, NVC with some degree of nasal notching has been reported in about 1%–21% of patients [2, 3, 4]. Of note, these studies have reported it to be subtle without requiring surgical repair. While NVC may be uncommon after MED, should it cause symptoms or aesthetic concerns, surgical correction would require suture lateralization or sidewall grafting with cadaveric or autologous rib cartilage, which could prove challenging. NVC should be less likely after MEDPAP, as the ligamentous attachments of the lower lateral cartilages should not be disturbed. This was supported by the 0% NVC rate in the current study and should be considered when considering MEDs.

Regarding crusting after MED approaches, presuming a substantial amount of MS mucosa is removed intraoperatively, patients should expect crusting that will usually resolve within 3 months. Fortunately, patients can always expect the MS to re‐mucosalize. However, in rare instances, crusting with or without purulence may persist and require ongoing topical irrigation with or without in‐office debridements.

A final theoretical risk with EMMs involving inferior turbinectomy would be ENS. Using the validated ENS6Q [19], no patients in the current study developed ENS based on all patients scoring less than 10.5 at final follow‐up. This is in line with two other studies of patients who had undergone subtotal inferior turbinectomies during endoscopic medial maxillectomies, with no patients developing elevated ENS6Q scores [20] or ENS manifestations [21]. These findings would also support conclusions from Kanjanawasee et al. that ENS is not caused simply by a relative increase in intranasal volume and that further research is necessary to understand ENS pathophysiology [22].

Taken together, MEDPAP compared favorably with prior MED reports with regard to complications and tumor recurrence rates. When compared to the largest MED studies reporting dentofacial hypesthesia sites [2, 4], MEDPAP resulted in roughly half the rate of long‐term dental hypesthesia. Additionally, there were no instances of NVC in the current study, while Bertazzoni et al., McCormick et al., and Turri‐Zanoni et al. reported one or more patients developing external NVC with classic MED approaches [2, 3, 4]. Considering the current study's low complication rates and 0% tumor recurrence rate, if MED is to be performed, surgeons should consider preserving the PA. One potential downside to PA preservation during MEDs is need to use angled nasal endoscopes for anterolateral MS dissection. Whether this is a barrier to adopting this MED modification will depend on surgeon comfort with angled endoscopes.

Limitations of this study should also be discussed. First, the study's generalizability was limited by the single‐center design and a relatively small sample size, albeit a comparable size to some of the largest MED‐related studies to date. Next, the small sample sizes of long‐term epiphora and hypesthesia prevented assessment for any associations between these complications and different tumor or hyperostotic origins. Another issue pertained to the reporting of times to resolution for hypesthesia, epiphora, and crusting. While they were assessed at clinic visits, times to hypesthesia and epiphora resolution were based on patient reporting, whereas crusting was based on the in‐office nasal endoscopy. Therefore, hypesthesia and epiphora resolution could have been affected by recall bias, and time to crusting resolution could have been overestimated. Regardless, the times to resolution of these complications were good estimates and can help with patient expectations. One other limitation pertaining to time to crusting resolution was that the extent of MS mucosal removal was not prospectively measured in each case. Whether crusting would resolve faster with less mucosal removal cannot be known from this study, though it is a reasonable presumption. In benign conditions, surgeons should aim to remove the least amount of normal mucosa that still ensures removal of the disease process. Lastly, the presence of infectious sinusitis was not collected prospectively. Some patients after MED can develop purulent sinusitis, and while it is usually manageable with oral and/or topical antimicrobial therapy, it is possible some patients could develop recalcitrant infectious rhinosinusitis. Future studies would be beneficial in determining how often infectious rhinosinusitis occurs following MED and MEDPAP and whether it becomes recalcitrant in some patients.

## 5 Conclusions

MEDPAP was 100% successful at managing benign and select malignant MS pathologies during the study period. Patients frequently experienced temporary epiphora (35%) and dentofacial hypesthesia or numbness (65%), but these symptoms resolved by 2‐6 months postoperatively. Epiphora and hypesthesia or numbness persisted in 7.7% and 12% of patients, respectively. No patients developed NVC or ENS.

## Conflicts of Interest

John R. Craig served as research consultant for Aerin Medical, Inc. The remaining authors have no conflicts of interest.

## Supporting information




**Supplementary File1**: alr70200‐sup‐0001‐FigureS1.jpg

**Supplementary File2**: alr70200‐sup‐0001‐TablesS1‐S3.docx
