# Supplementary material for: Complications and Clinical Outcomes Following Modified Endoscopic Denker's With Pyriform Aperture Preservation: A Prospective Analysis
Source: Int Forum Allergy Rhinol. 2026 Jun 12;16(11):1260–8. doi: 10.1002/alr.70200 (PMC13731150; doi:10.1002/alr.70200)
Supplement: Supplementary file 1 [file ALR-16-1260-s001.docx]

**Supplemental Tables**

**Supplemental Table 1**

| **Symptoms** | **Present at First Follow-up** | **Present at Final**  **Follow-up** | **Time to Resolution**  **[Days; Median (IQR)]** |
| --- | --- | --- | --- |
| Epiphora (n=52) | 18 | 4 | 63.0 (36.8, 127.7) |
| Crusting (n=52) | 46 | 1 | 91.0 (65.0, 189.0) |
| Hypesthesia/  Numbness (n=50) |  |  |  |
| Second Molar | 12 | 0 | 89.5 (73.0, 187.8) |
| First Molar | 13 | 0 | 91.0 (77.0, 167.0) |
| First Premolar | 18 | 2 | 100.5 (60.0, 187.8) |
| Nasal | 11 | 0 | 103.0 (63.0, 143.0) |
| Cheek | 15 | 0 | 110.0 (85.5, 336.5) |
| Upper Lip | 21 | 1 | 117.0 (84.0, 176.0) |
| Second Premolar | 14 | 0 | 120.5 (86.5, 363.2) |
| Lateral Incisor | 25 | 6 | 146.0 (52.5, 282.0) |
| Canine | 22 | 5 | 156.0 (61.0, 208.0) |
| Central Incisor | 23 | 4 | 167.0 (74.5, 310.5) |

Samples sizes of those who had epiphora, sinonasal crusting, and hypesthesia/numbness at first and final follow-ups, and median times to resolution of each complication in those whose complications resolved.

**Supplemental Table 2**

| **Outcomes** | **Early Cohort (N)** | **Late Cohort**  **(N)** | **Frequency in Early Cohort**  **[X/N (%)]** | **Frequency in Late Cohort**  **[X/N (%)]** | **p-value** |
| --- | --- | --- | --- | --- | --- |
| **Initial Postoperative Follow-up** | | | | | |
| Hypesthesia or Numbness (n=50) |  |  |  |  |  |
| Cheek | 24 | 26 | 8 (33.3) | 7 (26.9) | 0.760 |
| Lateral nasal  sidewall | 24 | 26 | 6 (25.0) | 5 (19.2) | 0.738 |
| Upper lip | 24 | 26 | 12 (50.0) | 9 (34.6) | 0.391 |
| Central  incisor | 24 | 26 | 12 (50.0) | 11 (42.3) | 0.777 |
| Lateral incisor | 24 | 26 | 13 (54.2) | 12 (46.2) | 0.778 |
| Canine | 24 | 26 | 10 (41.7) | 12 (46.2) | 0.783 |
| First  premolar | 24 | 26 | 10 (41.7) | 8 (30.8) | 0.557 |
| Second  premolar | 24 | 26 | 8 (33.3) | 6 (23.1) | 0.533 |
| First molar | 24 | 26 | 7 (29.2) | 6 (23.1) | 0.751 |
| Second molar | 24 | 26 | 6 (25.0) | 6 (23.1) | 1.000 |
| Epiphora  (n=52) | 26 | 26 | 10 (38.5) | 6 (23.1) | 0.368 |
| Crusting  (n=50) | 24 | 26 | 20 (83.3) | 24 (92.3) | 0.409 |
| **Final Postoperative Follow-up** | | | | | |
| Hypesthesia or Numbness (n=50) |  |  |  |  |  |
| Cheek | 24 | 26 | 0 (0.0) | 0 (0.0) | N/A |
| Lateral nasal  sidewall | 24 | 26 | 0 (0.0) | 0 (0.0) | N/A |
| Upper lip | 24 | 26 | 0 (0.0) | 1 (3.8) | 1.000 |
| Central  incisor | 24 | 26 | 2 (8.3) | 2 (7.7) | 1.000 |
| Lateral incisor | 24 | 26 | 3 (12.5) | 3 (11.5) | 1.000 |
| Canine | 24 | 26 | 2 (8.3) | 3 (11.5) | 1.000 |
| First  premolar | 24 | 26 | 2 (8.3) | 0 (0.0) | 0.225 |
| Second  premolar | 24 | 26 | 0 (0.0) | 0 (0.0) | N/A |
| First molar | 24 | 26 | 0 (0.0) | 0 (0.0) | N/A |
| Second molar | 24 | 26 | 0 (0.0) | 0 (0.0) | N/A |
| Epiphora  (n=52) | 26 | 26 | 2 (7.7) | 2 (7.7) | 1.000 |
| Crusting  (n=50) | 24 | 26 | 1 (4.2) | 0 (0.0) | 0.480 |

Comparisons of rates of complications between the first half (early cohort) and last half (late cohort) of patients who underwent modified endoscopic Denker’s with pyriform aperture preservation. P-value was generated from the Fisher’s exact test.

**Supplemental Table 3**

| **Outcomes** | **Early Cohort (N)** | **Late Cohort**  **(N)** | **Frequency in Early Cohort**  **[median (IQR)]** | **Frequency in Late Cohort**  **[median (IQR)]** | **p-value** |
| --- | --- | --- | --- | --- | --- |
| **Initial Postoperative Follow-up** | | | | | |
| SNOT-22 change (postoperative – preoperative) | 19 | 22 | 0.0 (+13.0, -7.0) | -6.0 (+8.0, -23.0) | 0.255 |
| ENS6Q | 24 | 26 | 1.0 (0.0, 4.0) | 4.0 (2.0, 7.0) | **0.010** |
| **Final Postoperative Follow-up** | | | | | |
| SNOT-22 change (postoperative – preoperative) | 19 | 22 | -12.0 (-7.0, -40.0) | -13.0 (-2.0, -43.0) | 0.676 |
| ENS6Q | 24 | 26 | 0.5 (0.0, 2.0) | 0.5 (0.0, 1.0) | 0.802 |

Comparisons of 22-item sinonasal outcome test (SNOT-22) score changes (preoperative to initial or final postoperative scores), and the final postoperative 6-item empty nose syndrome questionnaire (ENS6Q) scores between the first half (early cohort) and last half (late cohort) of patients who underwent modified endoscopic Denker’s with pyriform aperture preservation. Note that a negative SNOT-22 change means a score reduction. P-value was generated from Wilcoxon rank sum test, and bold values represented statistical significance.

**Supplemental Figure 1**

Boxplots of initial and final postoperative 6-item empty nose syndrome questionnaire (ENS6Q) scores. Comparison of the initial and final median ENS6Q scores were significantly different (Wilcoxon rank sum test p<0.001).
